# Evidence-Based Critical Assessment of the Success Rate of Dental Implants in Smokers: An Umbrella Systematic Review

**DOI:** 10.7759/cureus.70067

**Published:** 2024-09-24

**Authors:** Ujjaini Banerjee, Pankaj Dhawan, Sapna Rani, Neha Jain

**Affiliations:** 1 Department of Prosthodontics, Manav Rachna Dental College, Faridabad, IND

**Keywords:** dental implants, implant failure, marginal bone loss, meta-analysis, osseointegration, smoking, systematic review

## Abstract

The aim of this umbrella systematic review is to evaluate the success rate and osseointegration of dental implants in patients with a smoking habit. Electronic database searches of PubMed/Medline, Google Scholar, ScienceDirect, Cochrane Library, and EBSCOhost were done from 2006 until August 2024, using medical subject headings (MeSH) terms. Systematic reviews and meta-analyses (SRMAs) investigating the association between smoking and the rate of success of dental implants based on dental implant failure, marginal bone loss, survival, and peri-implant parameters were deemed eligible. The Assessment of Multiple Systematic Reviews Tool 2 (AMSTAR 2) was used to assess the quality of the included SRMAs. Seventeen systematic reviews have been included. In the reviews, 10 studies included meta-analyses (MAs) that evaluated implant failure rate and marginal bone loss. According to the AMSTAR 2 checklist, one review was scored “high,” seven reviews were scored “moderate,” seven reviews were scored “low,” and two reviews were scored “critically low” quality. The strength of evidence suggests a low level of success and high rate of failure of osseointegration of dental implants in smokers. However, the influence of smoking on the rate of dental implant failure seems to be associated with various factors such as peri-implant inflammatory markers, post-operative infections, etc. Inherently, the conclusion of this review is based on a limited number of systematic reviews.

## Introduction and background

Smoking has been long known to cause and aggravate many serious illnesses over time. It has been shown in a previous study that smoking is the leading cause of lung-related diseases, lung cancers, myocardial events, and chronic heart diseases [1]. The use of tobacco, in any form, has been associated with a higher chance of developing severe periodontal diseases. For such a deleterious habit, dental and overall oral health is the first of many that are affected. Hence, dental implant treatment has become a fairly popular procedure to restore the function of the oral health of such affected patients.

Recent SRMAs have reported a higher risk of failure of implants in the included randomized controlled trials (RCTs) and case series. These failures can be mostly attributed to the clinical as well as surgical markers that have been observed to be compromised in smokers, such as gingival and peri-implant parameters [2]. It has also been demonstrated in a few recent research that post-surgical healing and healing after periodontal (non-surgical) procedures is less than satisfactory amongst smokers. The patients’ group including smokers respond differently and inadequately to the peri-implant disease therapy [3]. Recent SRMAs have collected their data from the existing RCTs, case series, and other types of research published over the years. These studies were synthesized and analyzed to provide authors with relevant information and evidence. However, with time, a towering amount of research has been produced on this specific connection between smokers and the effect of the habit on the success of dental implants. Researchers are now faced with an insurmountable task of sourcing, collecting, and analyzing the evidence under one platform.

Here, the methodological approach of an umbrella review (UR) brings this overwhelming quantity of evidence together. This helps authors gain a better perspective of the published systematic reviews and meta-analyses (SRMAs). URs also known as a “synthesis of reviews” help researchers collate, analyze, and produce reliable and robust results on previously professionally researched topics [4]. To provide researchers with succinct and laconic reports of the eligible secondary evidence on the matter, in a short timeframe, URs are an efficient approach. To provide significant results for this UR, the use of an appropriate assessment tool is paramount. The evidence reviewed by the tool will determine the level of strength of evidence of the included studies. For the qualitative analysis of systematic reviews (SRs), we have employed an assessment tool with 16 items that are described in the Assessment of Multiple Systematic Reviews Tool 2 (AMSTAR 2) [5-7].

## Review

Methodology

The protocol for this UR was registered in PROSPERO (CRD42024575748) and developed following the recommendations of the Preferred Reporting Items for Systematic Reviews and Meta-Analyses (PRISMA) 2020 guidelines [5]. The following UR was performed in three stages.

Search strategy

Databases, namely, PubMed/Medline, Google Scholar, ScienceDirect, Cochrane Library, and EBSCOhost, were comprehensively searched for literature on existing SRMAs from Jan 2006 till August 2024. In the first stage of the review, two independent reviewers (UB and SR) searched the databases independently and examined the titles and abstracts. After the removal of duplicates and critical screening by applying inclusion and exclusion criteria, full-text reviews were screened by three reviewers (UB, SR, and NJ). All selected SRMAs were evaluated by three authors (UB, SR, and NJ) and denoted as acceptable, or not, for the UR. Any disagreements that arose were resolved by consensus and resolved after discussion with the fourth reviewer (PD). The third stage involved qualitative analysis of included SRMAs with the help of an appropriate tool (AMSTAR 2) [6,7]. 

Search terms

The following are the medical subject headings (MeSH) terms used: “dental implants” (MeSH) OR “dental implant” (MeSH) OR “implantology” (MeSH) OR “oral implant” (MeSH) OR “smoking” (MeSH) AND “tobacco” (MeSH) OR “nicotine” (MeSH) OR “smoker” (MeSH) OR “non-smoker” (MeSH) AND “systematic review” (MeSH) OR “review” (MeSH) OR “implant failure” (MeSH) OR “implant failure rate” (MeSH) OR “implant success” (MeSH) OR “dental implant failure” (MeSH) OR “implant success rate” (MeSH) OR “success rate” (MeSH) AND “marginal bone loss” (MeSH) OR “post operative bone loss” (MeSH) OR “bone loss” (MeSH) OR “implant survival” (MeSH) OR “osseointegration” (MeSH) OR “osseo-integration” [MeSH] OR “bone healing” (MeSH) AND “cigarette smoking” (MeSH) OR “cigarette smoke” (MeSH).

Inclusion criteria and exclusion criteria

SRs alone and SRs with meta-analyses (MAs) presented within the time frame of 2006 to August 2024, written and published in English, were included in the present UR. Reviews that include success rate and osseointegration in their outcomes. Reviews that included qualitative and quantitative analysis of studies were included while literature reviews, scoping reviews, cohort studies, case reports, and case series were excluded. Any reviews presented before the year 2006 were excluded. Reviews with no definite outcomes and analysis were excluded. Reviews written and published in languages other than English were also excluded.

Research question

What is the strength of evidence in SRs regarding the success rate and successful osseointegration of dental implants in smokers?

PICOS for this umbrella review was as follows:

P - Patients who have received dental implants for missing tooth/teeth

I - Patients who have a chronic smoking habit

C - Patients who do not smoke

O - Success rate and osseointegration of dental implants

S - SRs with/without MAs

Data collection

The characteristics of included reviews were extracted by one author (UB) and entered in tables, which were checked for accuracy and trueness by another author (PD) independently. The chart included the authors’ name, year of publication, country, number of studies included, total number of implants studied, design of studies included, meta-analysis performed or not, p-value of included studies, outcomes evaluated, quality assessment tool used, and conclusions (Table 1).

**Table 1:** Characteristics of included reviews RCT - Randomised controlled trial, NR - Not reported

Author, year	Country	The number of studies included	Total number of implants	Study design of included studies	Meta-analysis performed	P-value	Outcomes evaluated	Quality assessment tool used	Conclusion
Moraschini et al. (2015) [8]	Rio de Janeiro, Brazil	15	25,130	Retrospective-10, prospective-5	Yes	<0.00001	Marginal bone loss and implant failure rates	Newcastle-Ottowa scale (NOS)	A statistically significant difference in marginal bone loss was found between the smoking group and the non-smoking group, in favour of the non-smoking group.
Alfadda (2018) [9]	Canada	10	3304	Prospective cohort studies-6, prospective studies-1, RCT-3	Yes	<0.001	Rate of dental implant failure, marginal bone loss	MOOSE (Meta-analysis of observational studies in epidemiology); STROBE (Strengthening the reporting of observational studies in epidemiology)	The available scientific evidence suggests that smoking is associated with significantly increased rates of implant failure and marginal bone loss. This can be attributed to some chemicals, which are found in tobacco, which reduce the vascularity of peri-implant tissues, which in turn compromise bone healing.
Mustapha et al. (2022) [10]	Malmo, Sweden	292	35,511	Retrospective studies	Yes	<0.001	Implant failure, marginal bone loss	Quality Assessment Tool of the National Institutes of Health	Implants placed in smokers present a 140.2% higher risk of failure than implants placed in non-smokers; The difference in implant failure between the groups was statistically significant for implants placed in the maxilla and the mandible (higher for smokers); The mean difference in MBL between the groups was statistically significant (higher for smokers); There was no clear influence of the follow-up time on the effect size (OR) and MBL mean difference between groups.
Chrcanovic et al. (2015) [11]	Malmo, Sweden	107	19,836	RCT-4, Case-control trials-16, prospective studies-16, retrospective analyses-71	Yes	<0.00001	Implant failure, postoperative infection, marginal bone loss	Newcastle-Ottowa scale (NOS)	The results of the present review should be interpreted with caution due to the presence of uncontrolled confounding factors in the included studies. Within the limitations of the existing investigations, the results of the present study suggest that the insertion of dental implants in smokers affects the implant failure rates, the incidence of postoperative infections, as well as the marginal bone loss.
Naseri et al. (2020) [12]	Isfahan, Iran	23	NR	Retrospective studies-16, clinical trial-1, prospective studies-6	Yes	<0.001	Implant failure in patients who smoked less than 10, more than 10, less than 20, and more than 20 cigarettes in a day	Newcastle-Ottowa scale (NOS)	The risk of implant failure was elevated with an increase in the number of cigarettes smoked per day.
Farronato et al. (2022) [2]	Varese, Italy	7	284	Prospective studies-7	No	NR	Peri-implantitis, peri-implant mucositis	Newcastle-Ottowa scale (NOS)	Implants placed in smokers present a 140.2% higher risk of failure than implants placed in non-smokers.
Akram et al. (2018) [13]	Perth, Western Australia	4	256	Retrospective studies	Yes	0.001	Peri-implant bone loss (PIBL), probing depth (PD), plaque index, and bleeding on probing	Newcastle-Ottowa scale (NOS)	Water pipe smoking has a detrimental effect on peri-implant health. Clinicians should instruct and advise patients about poor prognosis and peri-implant diseases caused by water pipe smoking.
Telleman et al. (2011) [14]	Groningen, The Netherlands	29	2611	RCT-1, prospective cohort study-28	No	>0.05	Implant failure rate	‘‘Quality assessment of a cohort study’’ and ‘‘quality assessment of a randomized clinical trial’’ developed by the Dutch Cochrane Centre	For studies that also included smokers, the failure rate was 0.008 compared with 0.004 found in studies that excluded smokers. There is fair evidence that short (<10mm) implants can be placed successfully in the partially edentulous patient, although with a tendency towards an increasing survival rate per implant length, and the prognosis may be better in the mandible of non-smoking patients.
Hinode et al. (2006) [15]	Japan	19	1122	Cohort -7, case-control-12	Yes	<0.05	Osseointegrated implant failure	NR	Implant failure was elevated with an increase in the number of cigarettes smoked per day.
Strietzel et al. (2007) [1]	Germany	35	16508	RCT-1, prospective-11, retrospective-21	Yes	(0.03 0.05	Implant failure in smokers	NR	The systematic review indicated significantly enhanced risks of biological complications among smokers. Five studies revealed no significant impact of smoking on the prognosis of implants with particle-blasted, acid-etched, or anodic, oxidized surfaces.
Ismail et al. (2021) [16]	Cairo, Egypt	9	3251	Prospective cohort studies-6, RCTs-3	No	0.002	Implant failure	ROB28, ROBINS-I9 Cochrane tools	Implant placement in smokers seems to be possible, in addition to periodontal therapy and strict oral hygiene that might increase the chances of success. Since the quality of evidence is deficient, results should be taken cautiously.
Turri et al. (2016) [17]	Gothenburg, Sweden	6	1399	Retrospective cross-sectional study-2, case-control cross-sectional study-2, cross-sectional study-2	No	<0.001	Probing pocket depth, bleeding on probing, marginal bone loss	Newcastle-Ottowa scale (NOS)	Data from existing studies point to smoking and diabetes as biologic-associated factors for peri-implantitis.
Chambrone et al. (2015) [18]	Brazil, South America	8	1011	Retrospective case series-5, prospective case series-2, RCT-1	No	0.0001	Implant loss, radiographic evaluation	Newcastle Ottawa Scale and the Cochrane Collaboration’s quality assessment tool	Although smoking was associated with implant failure in most of the individual studies and the overall meta-analysis, the detrimental effect of smoking was not confirmed when only prospective data were assessed (though there are very few prospective cohort studies focusing on the influence of smoking on grafted maxillary sinuses).
Akel (2019) [19]	Pennsylvania, USA	23	23,529	Retrospective-12, RCT-3, prospective-7	Yes	<0.05	Number of failed implants, peri-implant marginal bone loss	Cochrane RoB tool, Newcastle- Ottowa scale (NOS)	So, for patients who actively smoke, as in other periodontal oral surgical procedures, the clinical recommendation for a period of abstinence that at least covers the pre-surgical evaluation, initial therapy, definite implant treatment & immediate post-op phases remains relevant.
Ghanem et al. (2017) [20]	Pakistan	8	80	Case-control studies-8	Yes	0.014	Bone to implant contact (BIC), osseointegration	Critical Appraisal Skills Program (CASP) cohort study checklist.	From a clinical perspective, the detrimental effects of tobacco smoking (which results in the intake of a variety of toxic components) on osseointegration cannot be disregarded.
Al-Qamshaa et al. (2022) [21]	Riyadh	123	NR	RCT-16, prospective studies-10, prospective controlled clinical trials-10, retrospective observational studies-87	No	NR	Implant failure and Marginal Bone Loss	Quality Assessment Tool of the National Institutes of Health	Implants placed in smokers present a higher risk of failure than implants placed in non-smokers.
Caggiano et al. (2022) [22]	Italy	7	NR	Case control-2, cross-sectional-2, cohort-1, prospective-2	No	<0.05	Effect of smoking cessation on clinical, radiographic, and crevicular periodontal parameters around natural teeth.	ROBINS-I tool	Reported findings on clinical and crevicular periodontal parameters around natural teeth were contrasting when comparing ex-smokers to current and non-smokers and were in favor of nonsmokers.

Qualitative assessment

The AMSTAR 2 [6] criteria are a 16-item checklist that is used as an evaluation tool for SRMAs used to assess the quality of included SRs. The included reviews were appraised by three reviewers (UB, SR, and NJ) independently. Each review and meta-analysis received a score based on whether the answer was “yes,” “no” or “partial yes.” The SRs were scored as "critically low quality," "low quality," "moderate quality," and "high quality" based on the score received after assessment. Once the evaluation was completed by all reviewers independently, the acceptance of every study was decided by consensus among all reviewers. When required, disagreements were solved by a discussion with the fourth reviewer (PD).

Results

Included Studies

**Figure 1:**
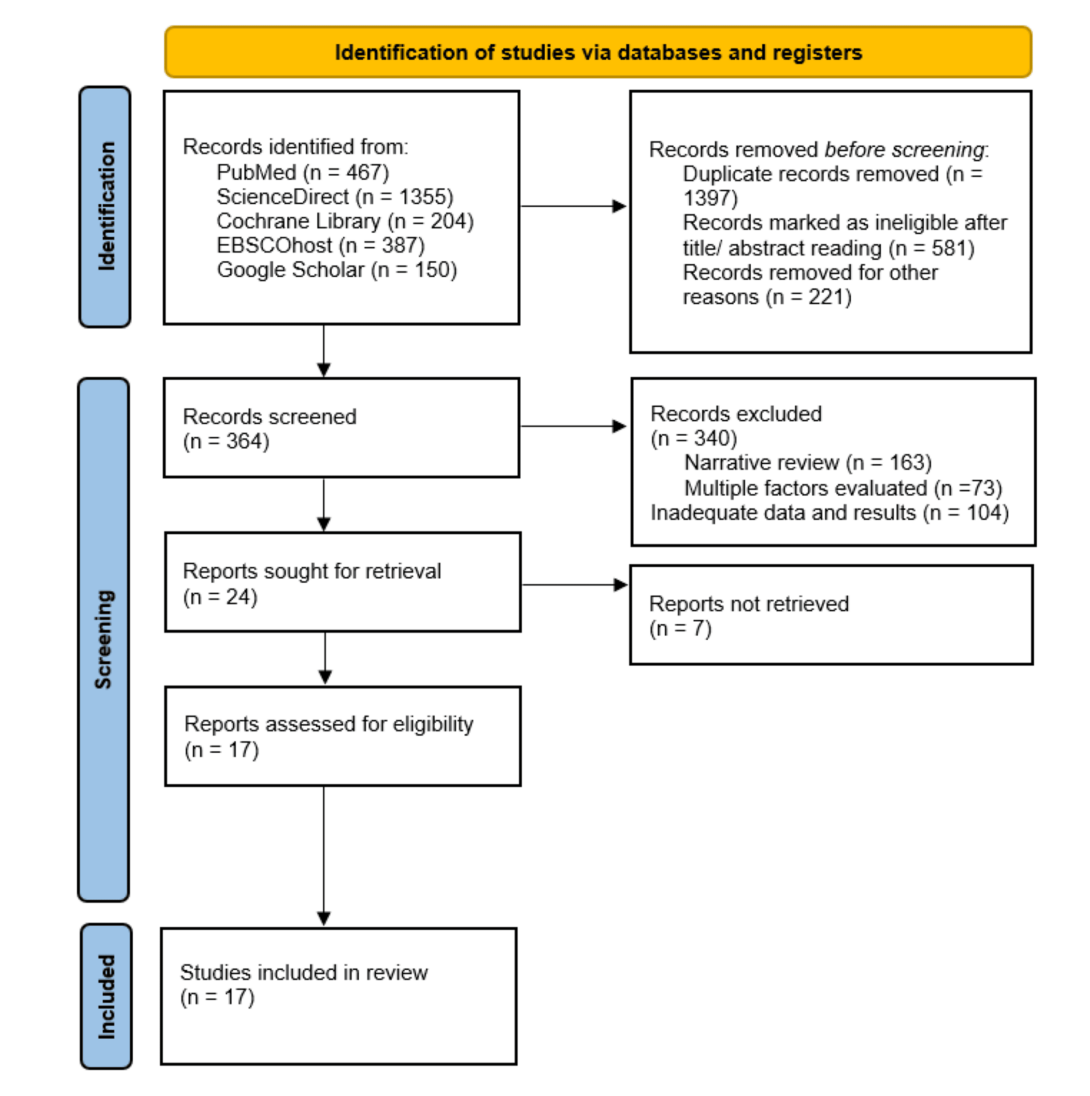
Flowchart of the selection process of the reviews A flowchart explaining the process of selection of reviews according to the Preferred Reporting Items for Systematic Reviews and Meta-Analyses (PRISMA) guidelines

The comprehensive literature search identified 2,563 SRMAs in the initial search. After screening these searches, reviews included after title/abstract reading and removing duplicates were 364 in total. According to the eligibility, 24 SRMAs, out of these 364 searches, met all the criteria for the present UR. However, full-text data were unavailable for seven reviews. Therefore, 17 reviews and 10 MAs were included in this umbrella review [1,2,4,7-20]. Figure 1 presents the PRISMA flowchart for the selection of the articles.

The attributes and main outcomes of the included SRs are presented with the help of Table 1. The included SRs were presented between 2006 and 2024. The total number of implants studied in each review was calculated. Outcomes assessed and p-values of the SR reviews were also included. The SRs have individually included a range of four to 292 studies. The study designs of the studies evaluated by the SRs ranged from retrospective, prospective to case-control, randomized controlled trials.

MAs were performed in 10 [1,4,7,8-11,13,17,18] out of the 17 SRs included. A summary of the MAs performed in the included studies has been given in Table 2.

**Table 2:** Summary of meta-analyses in the included studies Heterogeneity reported in individual reviews using the I² test, chi² test, and odds ratio (OR) included in the table.

Study	Outcome	P-value	Risk ratio	95% CI	Heterogeneity
Moraschini et al. (2015) [8]	Marginal bone loss between the maxilla and mandible in smokers, implant failure rate	< 0.00001	2.53	0.24-0.55	I² = 23.6%
Alfadda (2018) [9]	Rate of dental implant failure, marginal bone loss	< 0.001	2.92	1.76-4.83	I² = 65.8%
Mustapha et al. (2022) [10]	Implant failure, marginal bone loss	< 0.001	2.91	2.055-3.997	I² = 37.351%
Chrcanovic et al. (2015) [11]	Implant failure, postoperative infection, marginal bone loss	< 0.00001	2.23	1.96-2.93	I² = 51%
Naseri et al. (2020) [12]	Patients who smoked >20 cigarettes, >10 cigarettes, >15 cigarettes per day compared with non-smokers	< .001	2.45	1.42-4.22	I² = 71.0%
Akram et al. (2018) [13]	Peri-implant bone loss, probing depth between waterpipe smokers and non-smokers	0.001	2.49	1.01-3.97	I² = 94.16%
Hinode et al. (2006) [15]	Osseointegrated implant failure	< 0.05	2.17	1.67-2.83	OR = 2.17
Strietzel et al. (2007) [1]	Implant failure in smokers	0.013	2.38	1.93-2.93	OR = 2.25
Chambrone et al. (2015) [18]	Implant loss, radiographic evaluation	0.0001	1.87	1.35-2.58	Chi² = 2.32
Akel (2019) [19]	Implant failure rate, peri-implant marginal bone loss	< 0.00001	2.24	1.9-2.64	I² = 97%

The qualitative assessment of included SRs has been tabularized in Table 3.

**Table 3:** Systematic Reviews and Meta-Analyses (AMSTAR 2) score for the included SRMAs Scores denoted to each variable are as follows: partial yes (score: 0.5); yes (score: 1); no, no MA (no meta-analysis conducted) (score: 0) Scores denoted to each category are as follows: critically low score (1-4); low score (5-8); moderate score (9-12); high (13-16); total score (16)

AMSTAR 2	Alfadda (2018) [9]	Mustapha et al. (2022) [10]	Chrcanovic et al. (2015) [11]	Naseri et al. (2020) [12]	Farronato et al. (2022) [2]	Akram et al. (2018) [13]	Telleman et al. (2011) [14]	Hinode et al. (2006) [15]	Strietzel et al. (2007) [1]	Ismail et al. (2021) [16]	Turri et al. (2016) [17]	Moraschini et al. (2015) [8]	Chambrone et al. (2015) [18]	Akel (2019) [19]	Ghanem et al. (2017) [20]	Al-Qamshaa et al. (2022) [21]	Caggiano et al. (2022) [22]
Did the research questions and inclusion criteria for the review include the components of PICO?	Yes	Yes	Yes	Yes	Yes	Yes	No	No	Yes	Yes	Yes	Yes	Yes	Yes	Yes	Yes	Yes
Did the report of the review contain an explicit statement that the review methods were established before the conduct of the review and did the report justify any significant deviations from the protocol?	Partial yes	Partial yes	Partial yes	Partial yes	Partial yes	Partial yes	Yes	No	Yes	Yes	Partial yes	Yes	Partial yes	Yes	Yes	Partial yes	Partial yes
Did the review authors explain their selection of the study designs for inclusion in the review?	No	No	Yes	No	Yes	No	No	Yes	Yes	Yes	Yes	Yes	No	Yes	Yes	No	Yes
Did the review authors use a comprehensive literature search strategy?	Partial yes	Partial yes	No	Partial yes	Partial yes	Partial yes	Partial yes	No	Partial yes	Partial yes	No	Yes	Partial yes	Yes	Partial yes	Partial yes	Partial yes
Did the review authors perform study selection in duplicate?	No	Yes	Yes	Yes	Yes	Yes	Yes	Yes	No	Yes	Yes	Yes	Yes	No	Yes	Yes	Yes
Did the review authors perform data extraction in duplicate?	No	Yes	No	No	Yes	Yes	Yes	No	Yes	Yes	No	Yes	Yes	No	Yes	Yes	Yes
Did the review authors provide a list of excluded studies and justify the exclusions?	No	No	No	No	No	No	Yes	No	No	Partial yes	No	Yes	No	Yes	Yes	No	Yes
Did the review authors describe the included studies in adequate detail?	Yes	Partial yes	Partial yes	No	Yes	Partial yes	Partial yes	No	Partial yes	Partial yes	Partial yes	Yes	Yes	Yes	Partial yes	Partial yes	Partial yes
Did the review authors use a satisfactory technique for assessing the risk of bias (RoB) in individual studies that were included in the review?	Partial yes	Partial yes	Partial yes	Partial yes	No	Partial yes	Yes	No	No	Partial yes	Partial yes	Yes	No	Yes	Partial yes	Partial yes	Partial yes
Did the review authors report on the sources of funding for the studies included in the review?	Yes	No	No	No	No	Yes	Yes	No	Yes	No	No	Yes	Yes	No	No	No	No
If meta-analysis was performed did the review authors use appropriate methods for statistical combination of results?	Yes	Yes	Yes	Yes	No	Yes	No MA	Yes	Yes	Yes	No MA	Yes	Yes	Yes	Yes	No MA	No MA
If meta-analysis was performed, did the review authors assess the potential impact of RoB in individual studies on the results of the meta-analysis or other evidence synthesis?	No	Yes	No	No	No MA	No	No MA	No	Yes	Yes	No MA	Yes	Yes	Yes	Yes	No MA	No MA
Did the review authors account for RoB in individual studies when interpreting/discussing the results of the review?	Yes	Yes	No	No	Yes	No	No	No	Yes	Yes	No	Yes	Yes	Yes	Yes	No	Yes
Did the review authors provide a satisfactory explanation for, and discussion of, any heterogeneity observed in the results of the review?	Yes	Yes	Yes	Yes	No	No	No	Yes	Yes	Yes	No	Yes	Yes	Yes	Yes	No	No
If they performed quantitative synthesis did the review authors carry out an adequate investigation of publication bias (small study bias) and discuss its likely impact on the results of the review?	Yes	Yes	Yes	Yes	No MA	No	No MA	No	Yes	Yes	No MA	Yes	Yes	Yes	No	No MA	No MA
Did the review authors report any potential sources of conflict of interest, including any funding they received for conducting the review?	No	Yes	No	Yes	Yes	No	No	No	Yes	No	No	Yes	Yes	No	No	No	No
TOTAL SCORE	8	11	7.5	7.5	9	7	7	4	12	12	4.5	16	12	12	11.5	5	8
Level of Strength	Low	Moderate	Low	Low	Moderate	Low	Low	Critically low	Moderate	Moderate	Critically low	High	Moderate	Moderate	Moderate	Low	Low

Amongst the included reviews, one was scored of "high quality" [8], seven were scored of "moderate quality" [1,2,10,16,18-20], seven were scored of "low quality" [9,11-14,21,22], and lastly two were scored of "critically low" quality [15,17]. Here, the scores were divvied out of a total score of 16. The responses were given scores as "yes" equaled one mark, "no" equaled zero mark, and "partial yes" equaled half a mark. Ranges of marks between one to four were marked "critically low," five to eight marks were marked "low," nine to 12 marks were marked "moderate," and 13-16 marks were marked "high" quality. A tally of the scores has been presented tabularly in Table 3, which helped us evaluate the level of strength of evidence of the topic at hand. From individual assessments, the level of strength of evidence can be inferred to be within the categories of "low," "mediocre," and "high." Therefore, the level of strength of evidence is "high" in one, "mediocre" in seven, and "low" in nine of the included studies.

Discussion

URs are structured, schematic, and systematic assessments of various SRMAs performed on a specific scientific topic or research. From a clinical standpoint, the key purpose of a UR is a meticulous, systematic, expository summary of multiple studies based on previously published SRMAs. Since the early 1980s, dental implants have become accessible to the general population, and therefore primary and secondary sources of evidence have recorded data about the risk of placement of implants in smokers. Due to this abundant availability of secondary evidence (SRMAs), an umbrella review will provide a significant perspective. The authors have evaluated the strength of evidence of the available studies in a qualitative approach and then summarized the relevant information to help researchers.

The included SRMAs collated their information from previously conducted RCTs, case-control studies, retrospective studies, prospective studies, cohort studies, cross-sectional studies, and retrospective case series. All the included reviews have analyzed various numbers of implants placed in different subjects. In these 17 reviews, the review authors had also conducted MAs, which helped in scoring their analyses accurately. Amongst the included 17 studies, 15 SRMAs have reported statistically significant results, and two reviews have not reported relevant p-values [2,21]. Quality assessment tools used for conducting SRs were not reported in two studies [1,15]. For evaluating the strength of evidence of the assimilated studies, the AMSTAR 2 quality assessment tool was used. AMSTAR 2 is a 16-item checklist consisting of straightforward and adequately designed questions to assess SRMAs. This tool is user-friendly and provides results in a concise and within a short timeframe [7].

The events that were evaluated in the included studies comprised peri-implant health, marginal bone loss, post-operative infection, lack of/low level of osseointegration of dental implants, rate of failure, and frequency of smoking. The results of radiographic crestal bone loss (CBL), bleeding on probing (Bop), probing depth (PD), and plaque index (PI) and analysis of peri-implant sulcular fluid parameters were investigated by Farronato et al. [2] in relevance to smoking. Among smokers and nonsmokers, all surgical and non-surgical treatments that were included had statistically significant and different outcomes [2,8]. The correlation between smoking and the clinical parameters of peri-implant disease reported the effects produced by the habit. The outcomes evaluated were bleeding on probing, implant probing depth, radiographic crestal bone loss, plaque index, gingival bleeding index, and peri-implant sulcular fluid. Several studies included by the authors report that smoking causes significant changes. For nonsmokers, the success rate with a bone loss greater than 5 mm was about 50%, while for smokers, it was less than 20% [2]. When ex-smokers and present non-smokers were compared regarding the impact of quitting smoking on clinical, radiographic, and gingival crevicular periodontal parameters surrounding natural teeth and dental implants, the findings reported were inadequate and did not show statistical significance. The causative involvement of smoking in the development of these disorders, which are microbially linked inflammations, has long been supported by studies demonstrating smoking's intrinsic chemical and mechanical ability to degrade periodontal health.

It has been found that crevicular proinflammatory biomarkers are important diagnostic, as well as prognostic tools in periodontal and peri-implant disease. Proinflammatory cytokines, such as tumour necrosis factor-alpha and interleukin-1b, are released by macrophages in response to lipopolysaccharides found in bacterial membranes. These macrophages can trigger osteoclastogenesis, which mostly leads to bone resorption along with fibroblast death, which in turn contributes to clinical attachment loss. Crevicular interleukin-1b and tumour becrosis factor-alpha levels were considerably greater in current smokers, particularly traditional tobacco smokers vs. e-cigarette smokers than in ex-smokers and non-smokers. Nicotine inhibited the gene expression of many enzymes, which play a significant role in osteoblast proliferation, differentiation, and apoptosis, which in turn led to effects on bone formation and remodeling. Compared to non-smokers, smokers have greater concentrations of free radicals and oxidative stress indicators, which may indirectly activate bone pro-resorption pathways by influencing osteoclast activity and differentiation. Additionally, smoking may have an impact on the RANKL-RANK-OPG pathway, a group of metabolic pathways that control osteoclast activity and proliferation. The procedure ultimately interferes with the bone-healing process [10].

Mustapha et al. [10] concluded that implants placed in smokers presented with higher rates of failure and marginal bone loss. The possible reason could be due to the effect of smoking toxins, which had deleterious effects on bone metabolism, osteogenesis, and angiogenesis. It has been demonstrated that smoking cigarettes suppresses several physiological and biochemical processes that disrupt angiogenesis. This, in turn, leads to an irregular blood supply to tissues, ultimately reducing remodeling and tissue healing. Furthermore, it has been shown that smoking cigarettes lowers the expression of angiogenic markers during the early stages of bone repair, which hinders bone healing. Biological indicators related to the peri-implant area, such as the bleeding index, depth of peri-implant pockets, and level of peri-implant mucosal inflammation, are often worse in smokers than in non-smokers. For implants positioned in the mandible or maxilla, there was a statistically significant difference in the failure rate between the groups. These are important factors in osseointegration and long-term maintenance of implants [10]. In the meta-analysis conducted by Naseri et al. [12], the implant failure rates between the smokers’ subgroups were compared considering the different number of cigarettes smoked per day, there was a statistically significant risk of implant failure (p = 0.001) among the smokers who consumed 10 cigarettes/day when compared to nonsmokers; however, there were no significant statistical differences when patients who consumed 15 were compared to patients who consumed 20 cigarettes/day [12]. Hinode et al. [15], Ismail et al. [16], Turri et al. [17], Chambrone et al. [18], Qamshaa et al. [21], and Caggiano et al. [22] revealed a significant relationship between smoking and the risk of osseointegrated implant failure and rise in peri-implant marginal bone loss [15-18,21,22]. The authors have advised that, in addition, periodontal therapy and strict oral hygiene could increase the chances of success of dental implants [16,17]. Moraschini et al. [8] observed that the failure rate among smokers was higher than among nonsmokers. However, the MA revealed that the rate of implant failure does not increase with longer follow-up times (p = 0.26). A statistically significant difference in peri-implant marginal bone loss was discovered between smokers and nonsmokers, favoring the nonsmoking group. However, a subgroup analysis of follow-up time found no significant increase in implant failure proportional to increased monitoring duration (p = 0.26) [8]. Strietzel et al. have reported weighed mean values (WMV) and standard deviations (SD) of percentage distributions of female and male patients (M) considering different observation periods (group 1: observation period 41 years; group 2: 41 and 45 years). The included studies have reported 57.0 WMV in females and 43.0 WMV in males (SD=1.80), group 2 reported 54.6 WMV in females and 45.3 WMV in males (SD=1.44) [1]. According to Akel [19], for patients who actively smoke and wish to undergo other periodontal and oral surgical procedures, the clinical recommendation for a period of abstinence that at least covers the pre-surgical evaluation, initial therapy, definite implant treatment, and immediate post-op phases is inherently relevant [19].

The results of this review indicate that, due to changes caused to the periodontal, peri-implant, and bone markers using "nicotine" and/or "smoking" cigarettes, the success of dental implants and the rate of osseointegration is compromised [14-19]. These results are pertinent to the researchers and clinicians now, as this situation requires further evidence-based and methodical research focusing on the connection between implants’ success rate and "smoking." Only then, the gap in the scientific literature can be mended with relevant research, which in turn can help the plan of action for treatment plans, including dental implants for all patients. This review’s results also indicate that there is a lack of high-strength evidence in existing SRMAs [9,11-15,17,21,22]. Within the scope of this review, only one high-quality SRMA [8] has been found, and seven mediocre strength of evidence studies were included [1,2,10,16,18-20]. In this review, we have included studies that have compared the success rates and bone loss parameters among ex-smokers and non-smokers. We have assessed the impact of frequency of smoking (number of cigarettes per day) on implant failure rate. However, in the included studies, limited data were found that reported a significant comparison among different genders. Multiple SRs did not include MAs. Hence, for future research, SRs should be conducted comparing the effects of smoking among different genders. MAs should also be conducted in the upcoming research to provide significant results. Therefore, clinicians and practitioners should adequately inform and prioritize the importance of smoking cessation to patients who are active smokers interested in dental implant treatment modality.

## Conclusions

Although smoking is known to be harmful to implant health, it has been found that levels of tumor necrosis factor-alpha and crevicular interleukin-1b were found to be significantly higher in patients with present smoking habits. In the reviews that were included, it was discovered that patients who smoke had a noticeably higher rate of implant failure. Nicotine affects bone formation and remodeling by suppressing the gene expression of numerous enzymes. Smokers have higher levels of oxidative stress markers and free radicals than non-smokers do. These factors can affect osteoclast development and activity, which in turn can trigger bone pro-resorption pathways. There was strong evidence that smokers had increased marginal bone loss, peri-implant inflammation, loss of osseointegration, and postoperative infection risk. It was also found that there was an exponentially higher risk of implant loss amongst smokers who consume ≥10 cigarettes in a day than nonsmokers. Therefore, smoking is not an absolute contraindication for patients, but it would be prudent for clinicians to educate patients on smoking cessation and awareness regarding the same when dental implant treatment modality is necessary.
